# The associations between dietary flavonoid intake and hyperlipidemia: data from the national health and nutrition examination survey 2007–2010 and 2017–2018

**DOI:** 10.3389/fnut.2024.1374970

**Published:** 2024-05-31

**Authors:** Yingying Wan, Dan Ma, Linghua Yu, Wende Tian, Tongxin Wang, Xuanye Chen, Qinghua Shang, Hao Xu

**Affiliations:** ^1^National Clinical Research Center for Chinese Medicine Cardiology, Xiyuan Hospital, China Academy of Chinese Medical Sciences, Beijing, China; ^2^China Academy of Chinese Medical Sciences, Xiyuan Hospital Suzhou Hospital, Suzhou, China

**Keywords:** flavonoid, flavan-3-ol, anthocyanin, hyperlipidemia, NHANES

## Abstract

**Background:**

Hyperlipidemia is a worldwide health problem and a significant risk factor for cardiovascular diseases; therefore, it imposes a heavy burden on society and healthcare. It has been reported that flavonoids can increase energy expenditure and fat oxidation, be anti-inflammatory, and reduce lipid factor levels, which may reduce the risk of hyperlipidemia. However, the relationship between the prevalence of hyperlipidemia and dietary flavonoid intake in the population remains unclear.

**Methods:**

This study included 8,940 adults from the 2007–2010 and 2017–2018 National Health and Nutrition Examination Surveys (NHANES). The relationship between dietary flavonoid intake and the prevalence of hyperlipidemia was analyzed using weighted logistic regression and weighted restricted cubic spline.

**Results:**

We found an inverse relationship between subtotal catechins intake and hyperlipidemia prevalence in the third quartile [0.74 (0.56, 0.98), *p* = 0.04] compared with the first quartile. The prevalence of hyperlipidemia and total flavan-3-ol intake in the third quartile were inversely correlated [0.76 (0.59, 0.98), *p* = 0.03]. Total anthocyanin intake was inversely related to the prevalence of hyperlipidemia in the third quartile [0.77 (0.62, 0.95), *p* = 0.02] and the fourth quartile [0.77 (0.60, 0.98), *p* = 0.04]. The prevalence of hyperlipidemia was negatively correlated with total flavonols intake in the fourth quartile [0.75 (0.60, 0.94), *p* = 0.02]. Using restricted cubic splines analysis, we found that subtotal catechins intake and total flavan-3-ol intake had a nonlinear relationship with the prevalence of hyperlipidemia.

**Conclusion:**

Our study may provide preliminary research evidence for personalizing improved dietary habits to reduce the prevalence of hyperlipidemia.

## 1 Introduction

Hyperlipidemia usually refers to an increase in plasma triglycerides or total cholesterol, including an increase in low-density lipoprotein cholesterol (LDL-C) and a decrease in high-density lipoprotein cholesterol. Hyperlipidemia is a risk factor for atherosclerotic cardiovascular disease (1, 2). In practice, controlling LDL-C levels is the primary goal of hyperlipidemia treatment to reduce the prevalence and mortality of cardiovascular diseases (3, 4). Management of hyperlipidemia includes lifestyle interventions and pharmacotherapy (5, 6). Common lifestyle interventions include reducing the intake of saturated fatty acids and cholesterol, exercising regularly, controlling weight, quitting smoking, limiting alcohol intake, and limiting salt intake (7–10). Lipid regulators include medications that lower cholesterol, those that lower triglycerides, and newer lipid-lowering drugs (11–13). With aggressive, comprehensive management, the prognosis of hyperlipidemia is good. Patients with hyperlipidemia have elevated levels of lipids in their blood, which can lead to atherosclerosis, which in turn causes the narrowing of the coronary arteries and reduces blood flow to the heart. Long-term myocardial ischemia can cause angina pectoris and myocardial infarction, leading to a decline in cardiac function, which may eventually lead to heart failure, which is a major risk factor for coronary heart disease (14, 15). Therefore, active prevention and treatment are of great significance to reduce the incidence of cardiovascular disease and improve the quality of life (16).

Flavonoids are a large and diverse group of bioactive polyphenolic compounds found in plants (17). Flavonoids can be divided into six subclasses based on their chemical structures, including anthocyanins, flavan-3-ols, flavanones, flavones, flavonols, and isoflavones (18). In recent years, numerous studies have applied flavonoids and their metabolites to prevent and treat many diseases, including cancer, obesity, diabetes mellitus, hypertension, hyperlipidemia, cardiovascular disease, and osteoporosis (19, 20). Also, various studies have shown that some of the different flavonoids found in foods and herbs have anti-inflammatory, antioxidant, glycemic profile, and liver enzyme improvement effects (21–23). Previous studies have found that flavonoids can increase energy consumption and fat oxidation (24, 25), promote fat phagocytosis, reduce lipid factor levels, inhibit lipid accumulation in the liver, reverse liver function abnormalities caused by lipid peroxidation (26), and regulate metabolism and gut flora (27, 28). Anthocyanins can reduce oxidized LDL-C levels (29). Flavonoids in grape derivatives can reduce plasma lipid levels. Drinking moderate amounts of red wine can reduce the oxidation of low-density lipoprotein and reduce endothelial toxicity caused by oxidized low-density lipoprotein molecules, thereby directly reducing the incidence of atherosclerotic disease (30). Catechin can increase energy consumption and fat oxidation (24). Marrein promotes fat autophagy by regulating the PI3K/AKT/mTOR pathway, thereby lowering lipids (26). These studies suggest that flavonoids have a protective role in developing hyperlipidemia.

Currently, no clinical studies report the relationship between dietary flavonoids and the prevalence of hyperlipidemia. Therefore, this study utilized publicly available data from the USDA Codex Flavonoid Value Database (flavonoid database, 2007–2010 and 2017–2018), Diet Facts in the United States (WWEIA), and NHANES to explore the relationship between flavonoid intake and the prevalence of hyperlipidemia in US adults aged ≥20 years.

## 2 Materials and methods

### 2.1 Study population

We collected data from the NHANES, a national population survey conducted by the National Center for Health Statistics (NCHS) in the US. It uses complex, multi-stage, and probability sampling techniques and is released on a 2-year cycle. It aims to investigate the nutritional and health status of the entire population in the United States (31). Information can be retrieved on the NHANES website.^1^ The NCHS Ethics Review Board approved the NHANES study protocol, and each participant signed an informed consent form.

**Figure 1:**
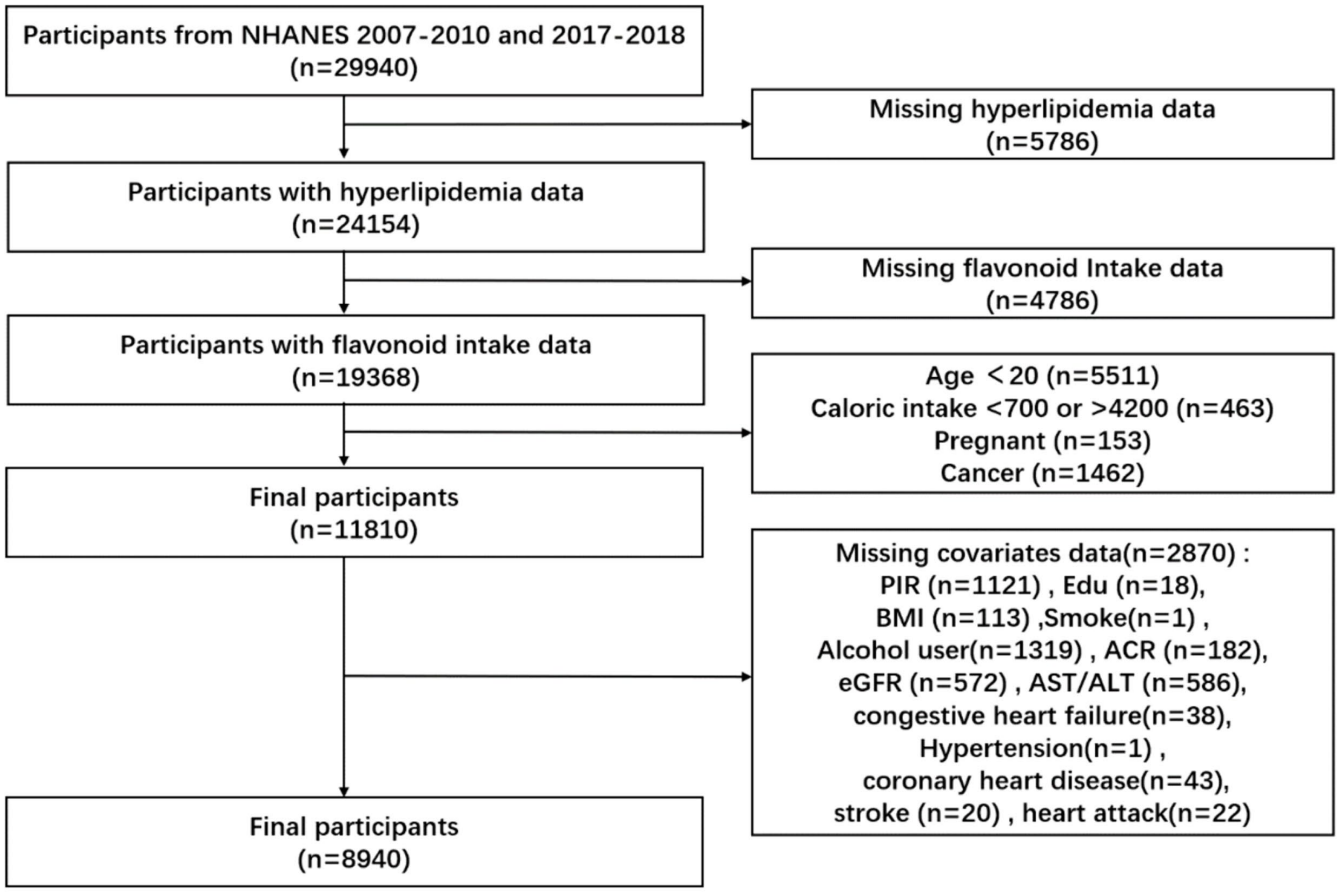
Flow chart of study participants.

We collected 29,940 participants from the NHANES database in consecutive NHANES cycles 2007–2010 and 2017–2018. We excluded 5,786 participants with missing data on a hyperlipidemia diagnosis, 4,786 participants with missing data on flavonoid intake, 5,511 participants younger than 20 years of age, 463 participants with caloric intake greater than 4,200 calories, or Participants with caloric intake less than 700 calories, 153 participants. Pregnant participants and 1,462 cancer participants, for a total of 8,940 participants (Figure 1).

### 2.2 Assessment of flavonoid intakes

We collected data on dietary flavonoid intake from the USDA Survey of Food and Beverage Flavonoid Values Database (“flavonoid database”). This database provides intakes of compounds from foods and beverages from the USDA Dietary Study Food and Nutrient Database (32) and corresponding dietary data from WWEIA (33) and NHANES. The USDA Nutrient Data Laboratory measured the content (mg/100 g) of 29 flavonoids in each food/beverage. Dietary flavonoids include the following seven flavonoid classes and the total daily intake of all flavonoids (the sum of 29 flavonoids) calculated from all foods and beverages. This study collected dietary flavonoid intake data from the flavonoid database from 2007–2010 to 2017–2018. We defined dietary flavonoid intake as the average of 2 days for each flavonoid.

### 2.3 Assessment of hyperlipidemia

Hyperlipidemia was identified when any of the following criteria were met: triglycerides ≥150 mg/dL; total cholesterol ≥200 mg/dL; low-density lipoprotein ≥130 mg/dL; high-density lipoprotein ≤40 mg/dL (male); high-density lipoprotein ≤50 mg/dL (female); or utilization of antihyperlipidemic agents.

### 2.4 Assessment of covariates

We included the following covariates: age, race, education, Family income-poverty ratio (PIR), body mass index (BMI), smoking status, alcohol drinking, caloric intake, Protein, Carbohydrate, total fat, total saturated fatty acids (total sfat), total polyunsaturated fatty acids (total mfat), total monounsaturated fatty acids (total pfat), total cholesterol, vitamin D, vitamin E, AST/ALT, ACR, eGFR, lipid-lowering drugs, hypertension, diabetes, heart attack, stroke, and coronary heart disease.

Participants were divided into the following three groups according to age: <30 years, 30–59 years, and ≥ 60 years. Race was divided into non-Hispanic White, non-Hispanic Black, Mexican-American, and others. The family income-poverty ratio was classified as <1.5, 1.5–3.5, and > 3.5. Education level was categorized as less than high school, high school or equivalent, some college or AA degree, and college graduate or above. Smoking status was classified as never (smoked fewer than 100 cigarettes in life), former (smoked more than 100 cigarettes in life and smoke not at all now), now (smoked more than 100 cigarettes in life and smoked some days or every day). Alcohol drinking was classified as never (had <12 drinks in a lifetime); former (had ≥12 drinks in 1 year and did not drink last year, or did not drink last year but drank ≥12 drinks in a lifetime); Mild (defined as two drinks per day for men and one drink per day for women); moderate (defined as three drinks per day for men and two drinks per day for women, or binge drinking 2–4 days per day); heavy (defined as ≥ four drinks per day for men and ≥ three drinks per day for women, or binge drinking ≥5 days per day) (34).

Disease covariates in this study include hypertension, diabetes, heart attack, stroke, and coronary heart disease. Based on the questionnaire and physical examination results, participants were diagnosed with hypertension if they met one of the following three conditions: (1) the average systolic blood pressure ≥ 130 mmHg or the average diastolic blood pressure ≥ 80 mmHg; (2) the answer to the question “have you ever been told to take a prescription for hypertension” was “yes”; (3) the answer to the question “have you ever been told that you had high blood pressure” was “yes.” All blood pressure determinations (systolic and diastolic) were taken at a mobile examination center. The following protocol calculated average blood pressure: The diastolic reading with zero was not used to calculate the diastolic average. If all diastolic readings were zero, then the average would be zero. If only one blood pressure reading was obtained, that reading is the average. If there was more than one blood pressure reading, the first reading was excluded from the average. The diagnostic criteria for diabetes were as follows: the doctor told the patient they have diabetes, HbA1c ≥ 6.5%; fasting glucose ≥7.0 mmol/L; random blood glucose ≥11.1 mmol/L; two-hour OGTT blood glucose ≥11.1 mmol/L; utilization of diabetes medication or insulin. DM: diabetes mellitus; IFG: impaired fasting glycemia (fasting glucose 6.1–7.0 mmol/L); IGT: Impaired Glucose Tolerance (two-hour OGTT blood glucose 7.8–11.1 mmol/L). Cardiovascular disease was diagnosed based on whether questionnaires and physical examination results were used, and cardiovascular disease was defined as heart attack, stroke, and coronary heart disease. The diagnostic criteria for heart attack: the answer to the question “Have you ever been told that you had a heart attack?” was “yes.” The diagnostic criteria for stroke: the answer to the question “Have you ever been told that (the patient) had a stroke?” was “yes.” The diagnostic criteria for coronary heart disease: the answer to the question “Have you ever been told that you had coronary heart disease?” was “yes.”

### 2.5 Statistical analysis

All statistical analyses were performed using R software (version 4.1.3). Data preparation and statistical analysis were performed using the R packages “*NHANESR*” and “*survey*.” In the analysis of baseline information, continuous variables were expressed as weighted means ± standard deviations using one-way analysis of variance to compare differences between groups; categorical variables were expressed as frequencies and percentages and compared using the chi-square test. We used four weighted logistic regression models to examine the relationship between flavonoid consumption and hypertension prevalence. The crude model was unadjusted. Model 1 was adjusted for age, race, and sex. Model 2 was adjusted for age, race, sex, caloric intake, smoking status, alcohol drinking, and PIR. Model 3 was adjusted for age, race, sex, caloric intake, smoking status, alcohol drinking, education, PIR, protein, total fat, total sfat, total mfat, total pfat, total cholesterol, vitamin D, vitamin E, ACR, eGFR, AST/ALT, lipid-lowering drugs, hypertension, heart attack, stroke, coronary heart disease, and diabetes.

We used weighted restricted cubic splines from the “*rms*” package to evaluate potential nonlinear associations. Subgroup weighted logistic regression was used to analyze the effect of flavonoid intake on the prevalence of hyperlipidemia, stratified by age, race, sex, caloric intake, smoking status, alcohol drinking, education, PIR, protein, total fat, total sfat, total mfat, total pfat, total cholesterol, vitamin D, vitamin E, ACR, eGFR, AST/ALT, lipid-lowering drugs, hypertension, heart attack, stroke, coronary heart disease, and diabetes. Weighted logistic regression was used to calculate odds ratios and corresponding 95% confidence intervals. A significance level of *p* < 0.05 was used as the threshold for statistical significance. The P for interaction was based on the log-likelihood ratio test to assess the heterogeneity of the relationship between subgroups.

## 3 Results

### 3.1 Characteristics of participants

We included 8,940 participants. The baseline characteristics were grouped according to hyperlipidemia (Table 1). There were 6,520 patients with hyperlipidemia and 2,420 without. The average age of healthy participants was 38.89 (0.51) years, and that of participants with hyperlipidemia was 48.90 (0.37) years. Non-Hispanic white, Mexican-American participants and participants in less than high school and high school or equivalent were more likely to have hyperlipidemia than healthy participants. In terms of smoking status, more participants with hyperlipidemia were current or former smokers than healthy participants. Among participants with hyperlipidemia, the proportion of former or mild drinkers was higher than that of healthy participants. Participants with hyperlipidemia had a higher BMI, lower caloric intake, lower total pfat intake, and lower vitamin E intake than healthy participants. Hyperlipidemic participants had lower ACR, higher eGFR, and lower AST/ALT compared to healthy participants. Among 8,940 participants, 1753 participants took lipid-lowering medications and 7,187 participants did not take lipid-lowering medications (Supplementary Table 1). Among the 6,520 participants with hyperlipidemia, 1753 participants took lipid-lowering drugs and 4,767 participants did not take lipid-lowering drugs. In terms of comorbidities, participants with hyperlipidemia had higher rates of hypertension, diabetes, stroke, heart attack, and coronary heart disease than healthy participants. There were no significant differences in sex, PIR, protein, total fat, total sfat, total mfat, total cholesterol, and vitamin D between healthy participants and those with hyperlipidemia. However, the participants with hyperlipidemia had a higher intake of subtotal catechins, total flavan-3-ols, and total flavonoids. The specific content of participants’ flavonoid dietary assessment is provided in Supplementary Table 2.

**Table 1:** Characteristics of participants in the study.

Variable	Participants without hyperlipidemia	Participants with hyperlipidemia	*p-*value
N	2,420	6,520	
Age (years)			< 0.0001
20–39	2,998(44.86)	54 (4.00)	
40–59	2,664 (40.55)	503 (38.72)	
≥60	1,525 (14.59)	1,196 (57.28)	
Sex, N (%)			0.42
Female	1,228 (51.40)	3,410 (52.97)	
Male	1,192 (48.60)	3,110 (47.03)	
Race, N (%)			< 0.0001
Non-Hispanic White	1,009 (65.30)	3,114 (70.98)	
Non-Hispanic Black	595 (12.74)	1,130 (9.21)	
Mexican American	359 (7.86)	1,134 (8.56)	
Others	457 (14.10)	1,142 (11.25)	
Education, N (%)			< 0.0001
Less than high school	461 (11.35)	1,644 (15.44)	
High school or equivalent	508 (22.04)	1,579 (26.44)	
Some college or AA degree	782 (31.74)	1915 (30.82)	
College graduate or above	669 (34.87)	1,382 (27.31)	
Smoking status, N (%)			< 0.0001
Now	458 (16.67)	1,351 (19.75)	
Former	459 (20.71)	1,681 (24.94)	
Never	1,503 (62.62)	3,488 (55.31)	
Alcohol drinking, N (%)			< 0.0001
Former	248 (6.96)	1,026 (12.46)	
Heavy	609 (27.19)	1,314 (20.54)	
Mild	819 (36.11)	2,301 (39.31)	
Moderate	461 (19.91)	1,042 (18.15)	
Never	283 (9.84)	837 (9.54)	
PIR, N (%)			0.95
<1.5	805 (23.66)	2,331 (24.06)	
1.5–3.5	803 (30.10)	2,129 (30.19)	
>3.5	812 (46.24)	2060 (45.75)	
BMI (kg/m^2^)	26.69 (0.20)	30.14 (0.13)	< 0.0001
Caloric intake (kcal)	2105.57 (19.50)	2055.48 (14.98)	0.03
Protein (g)	83.31 (0.97)	81.16 (0.70)	0.05
Carbohydrate (g)	249.07 (2.67)	244.59 (1.65)	0.13
Total fat (g)	80.78 (1.03)	79.70 (0.79)	0.4
Total sfat (g)	26.16 (0.38)	26.17 (0.28)	0.97
Total mfat (g)	28.64 (0.37)	28.45 (0.29)	0.68
Total pfat (g)	18.53 (0.28)	17.74 (0.22)	0.04
Total cholesterol (mg)	280.22 (4.07)	288.25 (4.20)	0.14
Vitamin D (mcg)	4.51 (0.10)	4.57 (0.08)	0.63
Vitamin E (mg)	8.83 (0.21)	7.97 (0.13)	< 0.001
ACR (mg/g)	18.03 (2.27)	31.55 (3.48)	0.01
eGFR (ml/min/1.73m^2^)	102.41 (0.72)	93.01 (0.52)	< 0.0001
AST/ALT	1.19 (0.01)	1.07 (0.01)	< 0.0001
Lipid-lowering drugs, N (%)			< 0.0001
No	2,420 (100.00)	4,767 (76.55)	
Yes	0 (0.00)	1753 (23.45)	
Hypertension, N (%)			< 0.0001
No	1847 (80.75)	3,619 (60.49)	
Yes	573 (19.25)	2,901 (39.51)	
Stroke			< 0.0001
No	2,381 (98.74)	6,243 (96.81)	
Yes	39 (1.26)	277 (3.19)	
Heart attack, N (%)			< 0.0001
No	2,379 (98.92)	6,228 (96.56)	
Yes	41 (1.08)	292 (3.44)	
Coronary heart disease, N (%)			< 0.0001
No	2,395 (99.38)	6,219 (96.00)	
Yes	25 (0.62)	301 (4.00)	
Diabetes, N (%)			< 0.0001
No	2078 (90.27)	4,506 (75.24)	
DM	93 (2.96)	374 (5.53)	
IFG	188 (4.74)	1,390 (15.90)	
IGT	61 (2.03)	250 (3.33)	
Dietary intake of flavonoids (mg/day)			
Subtotal Catechins	67.31 (3.83)	82.10 (5.37)	0.02
Total Isoflavones	3.23 (0.36)	1.74 (0.22)	0.001
Total Anthocyanidins	14.46 (1.42)	14.35 (0.98)	0.94
Total Flavan-3-ols	151.81 (8.45)	184.18 (9.27)	0.002
Total Flavanones	12.69 (0.65)	12.20 (0.40)	0.43
Total Flavones	0.95 (0.04)	0.90 (0.03)	0.33
Total Flavonols	18.36 (0.36)	18.90 (0.42)	0.21
Total Sum of all 29 flavonoids	201.49 (8.99)	232.29 (9.57)	0.004

### 3.2 Associations between flavonoid intake and prevalence of hyperlipidemia

We analyzed weighted logistic regression to evaluate the potential association between flavonoid intake, and hyperlipidemia. Age, race, sex, caloric intake, smoking status, alcohol drinking, education, PIR, protein, total fat, total sfat, total mfat, total pfat, total cholesterol, vitamin D, vitamin E, ACR, eGFR, AST/ALT, lipid-lowering drugs, hypertension, heart attack, stroke, coronary heart disease, and diabetes were fully adjusted. We divided isoflavone intake into four groups based on flavonoid subclass intake; because more than 50% of participants did not report isoflavone intake, we divided isoflavone intake into two groups based on the median intake (Table 2).

**Table 2:** Associations between flavonoid intake and hyperlipidemia.

Flavonoid intake	Q1	Q2		Q3		Q4		
		OR (95% CI)	*p* value	OR (95% CI)	*p* value	OR (95% CI)	*p* value	*p* for trend
Total Sum of all 29 flavonoids(mg/day)	≤25.15	25.15–65.16		65.16–229.68		≥229.28		
Crude model	ref	0.80 (0.66,0.98)	0.03	0.90 (0.75,1.08)	0.24	0.85 (0.70,1.02)	0.08	0.42
Model 1	ref	0.70 (0.57,0.86)	0.001	0.71 (0.58,0.87)	0.002	0.69 (0.57,0.83)	<0.001	0.03
Model 2	ref	0.74 (0.61,0.90)	0.004	0.76 (0.62,0.93)	0.01	0.72 (0.60,0.85)	<0.001	0.04
Model 3	ref	0.83 (0.63,1.12)	0.07	0.82 (0.64,1.04)	0.09	0.86 (0.62,1.14)	0.12	0.18
Subtotal Catechins (mg/day)	≤4.96	4.96–14.80		14.80–68.86		≥68.86		
Crude model	ref	0.96 (0.76,1.22)	0.75	0.79 (0.62,1.00)	0.05	0.97 (0.80,1.19)	0.79	0.56
Model 1	ref	0.87 (0.67,1.11)	0.26	0.65 (0.50,0.84)	0.002	0.80 (0.64,0.99)	0.04	0.36
Model 2	ref	0.89 (0.69,1.14)	0.34	0.67 (0.52,0.88)	0.004	0.82 (0.66,1.01)	0.06	0.44
Model 3	ref	0.92 (0.70,1.22)	0.55	0.74 (0.56,0.98)	0.04	0.86 (0.68,1.09)	0.19	0.6
Total Isoflavones (mg/day)	≤0.01	0.01–366.18						
Crude model	ref	0.85 (0.73,0.99)	0.03					
Model 1	ref	0.86 (0.74,1.02)	0.08					
Model 2	ref	0.89 (0.76,1.05)	0.16					
Model 3	ref	0.99 (0.81,1.20)	0.87					
Total Anthocyanidins (mg/day)	≤0.14	0.14–2.05		2.05–11.20		≥11.20		
Crude model	ref	1.13 (0.89,1.42)	0.30	0.85 (0.71,1.04)	0.11	0.90 (0.73,1.11)	0.30	0.15
Model 1	ref	0.98 (0.78,1.24)	0.86	0.69 (0.57,0.84)	<0.001	0.63 (0.51,0.80)	<0.001	<0.001
Model 2	ref	1.00 (0.79,1.27)	0.99	0.72 (0.59,0.89)	0.003	0.69 (0.54,0.87)	0.003	0.002
Model 3	ref	1.04 (0.80,1.34)	0.76	0.77 (0.62,0.95)	0.02	0.77 (0.60,0.98)	0.04	0.04
Total Flavan-3-ols (mg/day)	≤5.04	5.04–15.55		15.55–165.22		≥165.22		
Crude model	ref	0.93 (0.74,1.18)	0.56	0.84 (0.68,1.03)	0.09	0.94 (0.76,1.17)	0.57	0.83
Model 1	ref	0.83 (0.64,1.07)	0.15	0.67 (0.53,0.85)	0.001	0.79 (0.63,0.99)	0.04	0.51
Model 2	ref	0.85 (0.66,1.09)	0.20	0.70 (0.56,0.88)	0.004	0.81 (0.65,1.01)	0.06	0.56
Model 3	ref	0.89 (0.68,1.17)	0.37	0.76 (0.59,0.98)	0.03	0.86 (0.67,1.12)	0.24	0.83
Total Flavanones (mg/day)	≤0.07	0.0–0.70		0.70–19.21		≥19.21		
Crude model	ref	0.89 (0.74,1.07)	0.21	0.95 (0.77,1.18)	0.65	0.92 (0.75,1.13)	0.41	0.66
Model 1	ref	0.83 (0.68,1.02)	0.08	0.84 (0.66,1.06)	0.14	0.77 (0.61,0.97)	0.03	0.05
Model 2	ref	0.87 (0.70,1.08)	0.21	0.90 (0.70,1.16)	0.40	0.82 (0.64,1.05)	0.10	0.12
Model 3	ref	0.89 (0.69,1.14)	0.31	0.95 (0.72,1.25)	0.68	0.84 (0.64,1.10)	0.19	0.17
Total Flavones (mg/day)	≤0.19	0.19–0.53		0.53–1.09		≥1.09		
Crude model	ref	0.92 (0.75,1.14)	0.45	0.94 (0.76,1.16)	0.53	0.83 (0.67,1.02)	0.08	0.11
Model 1	ref	0.86 (0.70,1.05)	0.14	0.80 (0.65,0.99)	0.04	0.67 (0.54,0.85)	0.001	0.002
Model 2	ref	0.88 (0.71,1.10)	0.25	0.84 (0.68,1.05)	0.12	0.72 (0.57,0.91)	0.01	0.01
Model 3	ref	0.85 (0.66,1.08)	0.16	0.83 (0.65,1.06)	0.13	0.79 (0.61,1.03)	0.08	0.13
Total Flavonols (mg/day)	≤7.17	7.17–13.09		13.09–22.74		≥22.74		
Crude model	ref	0.86 (0.72,1.01)	0.07	0.89 (0.73,1.08)	0.23	0.84 (0.68,1.03)	0.10	0.19
Model 1	ref	0.79 (0.66,0.95)	0.01	0.78 (0.64,0.96)	0.02	0.70 (0.57,0.85)	<0.001	0.003
Model 2	ref	0.81 (0.68,0.97)	0.02	0.81 (0.67,0.99)	0.04	0.71 (0.58,0.88)	0.002	0.01
Model 3	ref	0.87 (0.72,1.04)	0.12	0.85 (0.68,1.07)	0.15	0.75 (0.60,0.94)	0.02	0.02

Crude model: unadjusted. Model 1: adjusted by age, race, and sex. Model 2: adjusted by age, race, sex, caloric intake, smoking status, alcohol drinking, education, PIR. Model 3: adjusted by age, race, sex, caloric intake, smoking status, alcohol drinking, education, PIR, protein, total fat, total sfat, total mfat, total pfat, total cholesterol, vitamin D, vitamin E, ACR, eGFR, AST/ALT, lipid-lowering drugs, hypertension, heart attack, stroke, coronary heart disease, and diabetes.

In model 3, we observed that compared with the first quartile, there was an inverse relationship between subtotal catechins intake and the prevalence of hyperlipidemia in the third quartile [0.74 (0.56, 0.98), *p* = 0.04]. However, the *p*-value for the trend was insignificant (*p* = 0.6). Similarly, there was an inverse relationship between total flavan-3-ols intake and the prevalence of hyperlipidemia in the third quartile [0.76 (0.59, 0.98), *p* = 0.03], with a non-significant *p*-value for trend (*p* = 0.83). Compared with the first quartile, there was an inverse relationship between total anthocyanins intake and the prevalence of hyperlipidemia in the third [0.77 (0.62, 0.95), *p* = 0.02] and fourth quartiles [0.77 (0.60, 0.98), *p* = 0.04], the *p*-value for trend was significant (*p* = 0.04). Total flavonols intake in the fourth quartile [0.75 (0.60, 0.94), *p* = 0.02] was inversely related to the prevalence of hyperlipidemia, and the p-value for trend was significant (*p* = 0.02).

**Figure 2:**
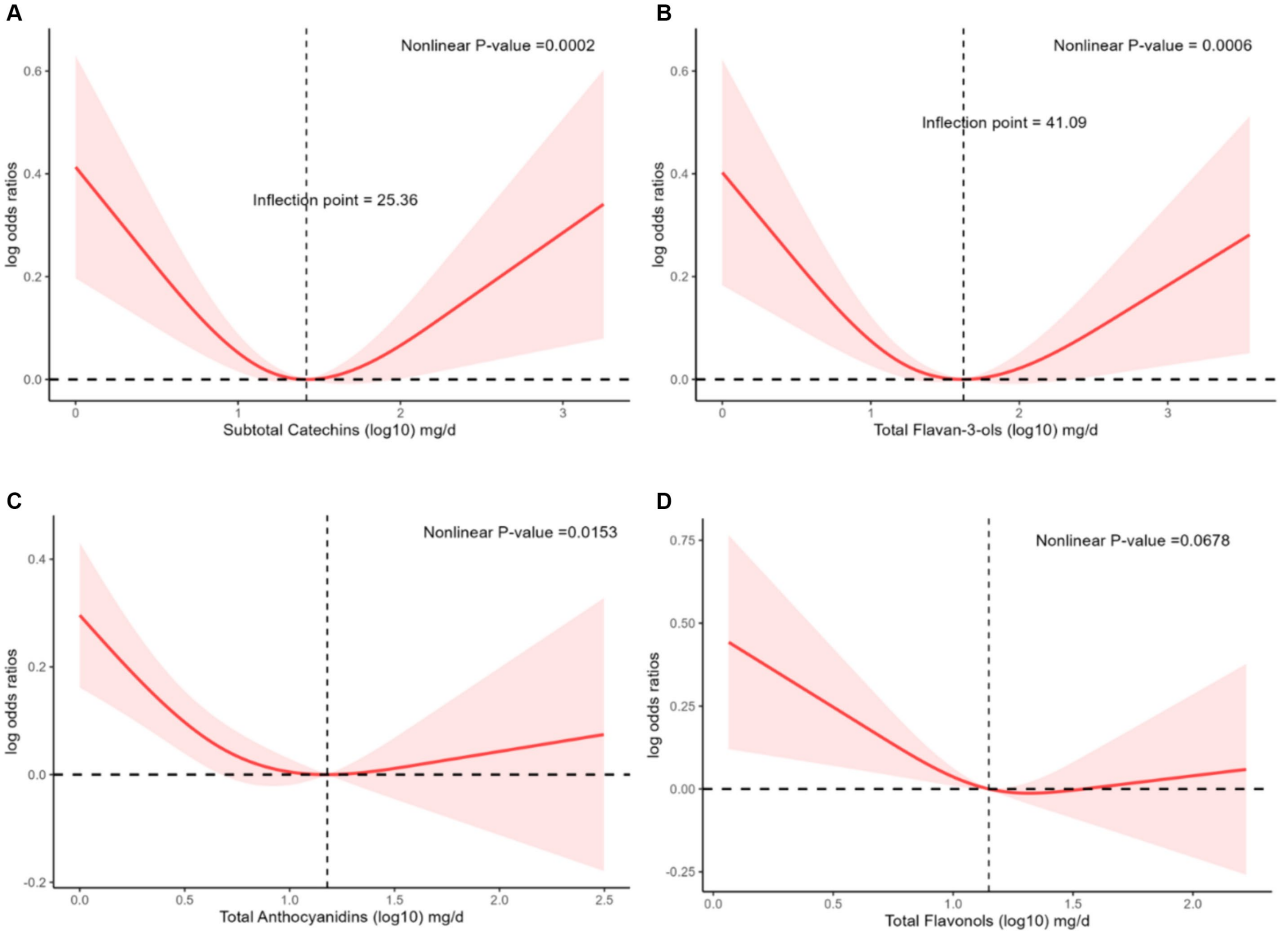
The association of flavonoid intake with prevalence of hyperlipidemia by restricted cubic splines. The y axis stands for the Log odds ratio of hyperlipidemia, and the X-axis stands for the log10 transformed intake of subtotal catechins **(A)**, total flavan-3-ols **(B)**, total anthocyanidins **(C)**, and total flavonols **(D)**. Models by restricted cubic splines were adjusted for age, race, sex, caloric intake, education, smoking status, alcohol drinking, PIR, protein, total fat, total sfat, total mfat, total pfat, total cholesterol, vitamin D, vitamin E, ACR, eGFR, AST/ALT, lipid-lowering drugs, hypertension, heart attack, stroke, coronary heart disease, and diabetes.

Because the p-value for a trend of the prevalence of hyperlipidemia and subtotal catechins intake and total flavan-3-ols intake were not significant, we considered a possible nonlinear relationship. We performed analyses using restricted cubic splines to explore whether there might be a nonlinear relationship between the prevalence of hyperlipidemia and subtotal catechins intake and total flavan-3-ols intake. There was a significant nonlinear relationship between the prevalence of hyperlipidemia and subtotal catechins intake (Figure 2A, *p* = 0.0002), total flavan-3-ols intake (Figure 2B, *p* = 0.0006), and total anthocyanidins (Figure 2C, *p* = 0.0153). In the above results, the nonlinear relationship between the prevalence of hyperlipidemia and subtotal catechins intake and total flavan-3-ols intake showed a U-shaped correlation. Through analysis, we observed that when the subtotal catechin intake was less than 25.36 mg/day, there was a significant negative linear relationship between the prevalence of hyperlipidemia and subtotal catechin intake. When the total flavan-3-ols intake is less than 41.09 mg/day, the prevalence of hyperlipidemia had a significant negative linear relationship with the total flavan-3-ols intake. However, the nonlinear relationship between the prevalence of hyperlipidemia and total flavones did not reach significance (Figure 2D).

### 3.3 Subgroup analysis

To assess the robustness of the association between flavonoid intake and hyperlipidemia， we performed subgroup analysis using weighted logistic regression to determine the subgroup interaction effect between flavonoid intake and the prevalence of hyperlipidemia. Stratified analyses were adjusted for age, sex, race, education, smoking status, alcohol drinking, PIR, caloric intake, hypertension, heart attack, stroke, coronary heart disease, diabetes, and other variables.

After analysis, we found that the interaction between the prevalence of hyperlipidemia and subtotal catechins intake (Table 3, *p* for interaction = 0.005) was significant when stratified by sex. However, when stratified by other variables, the relationship between the prevalence of hyperlipidemia and subtotal catechins intake was not statistically significant. This finding showed that age, race, education, smoking status, alcohol drinking, PIR, caloric intake, hypertension, heart attack, stroke, coronary heart disease, diabetes, and other variables did not significantly affect the relationship between the prevalence of hyperlipidemia and subtotal catechins intake (*p* for interaction >0.05). The interaction between the prevalence of hyperlipidemia and total flavan-3-ols intake was significant when stratified by sex (Supplementary Table 3, *p* for interaction = 0.01) and heart attack (Supplementary Table 3, *p* for interaction = 0.02). However, age, sex, race, education, smoking status, alcohol drinking, PIR, caloric intake, hypertension, heart attack, stroke, coronary heart disease, diabetes, and other variables did not significantly affect the relationship between the prevalence of hyperlipidemia and total anthocyanidin intake (Supplementary Table 4, *p* for interaction > 0.05). There was no significant interaction between hyperlipidemia prevalence and total flavonols intake when stratified by age, sex, race, education, smoking status, alcohol drinking, PIR, caloric intake, hypertension, heart attack, stroke, coronary heart disease, diabetes, and other variables (Supplementary Table 5, *p* for interaction >0.05).

**Table 3:** Subgroup analysis between hyperlipidemia and subtotal Catechins.

Variables	Q1	Q2		Q3		Q4			
		OR (95%CI)	*p* value	OR (95%CI)	*p* value	OR (95%CI)	*p* value	*p* for trend	*p* for interaction
Age									0.92
20–39	ref	0.93 (0.67,1.28)	0.65	0.85 (0.59,1.23)	0.39	0.97 (0.69,1.35)	0.83	0.87	
40–59	ref	1.00 (0.64,1.56)	0.99	0.67 (0.46,1.00)	0.05	0.82 (0.62,1.10)	0.18	0.46	
≥60	ref	0.90 (0.52,1.54)	0.69	0.68 (0.35,1.30)	0.23	0.80 (0.44,1.44)	0.44	0.75	
Race									0.51
Non-Hispanic White	ref	1.00 (0.69,1.46)	0.99	0.79 (0.55,1.12)	0.17	0.98 (0.73,1.30)	0.87	0.7	
Non-Hispanic Black	ref	1.13 (0.78,1.65)	0.50	1.33 (0.93,1.90)	0.12	1.33 (0.97,1.83)	0.07	0.12	
Mexican American	ref	0.75 (0.43,1.31)	0.30	0.83 (0.50,1.40)	0.48	0.72 (0.42,1.23)	0.22	0.41	
Others	ref	1.26 (0.75,2.14)	0.38	1.06 (0.61,1.84)	0.82	1.23 (0.74,2.03)	0.42	0.62	
Sex									0.005
Female	ref	0.83 (0.59,1.18)	0.30	0.84 (0.64,1.11)	0.22	1.20 (0.91,1.58)	0.20	0.01	
Male	ref	1.31 (0.98,1.76)	0.07	0.93 (0.64,1.34)	0.69	0.91 (0.71,1.17)	0.46	0.1	
Education									0.15
Less than high school	ref	0.85 (0.53,1.35)	0.47	1.21 (0.77,1.91)	0.40	1.10 (0.75,1.61)	0.64	0.52	
High school or equivalent	ref	0.90 (0.56,1.46)	0.67	0.55 (0.31,0.98)	0.04	1.10 (0.68,1.78)	0.68	0.24	
Some college or AA degree	ref	1.35 (0.96,1.92)	0.09	1.27 (0.95,1.70)	0.10	1.31 (0.93,1.85)	0.12	0.44	
College graduate or above	ref	1.02 (0.63,1.66)	0.92	0.86 (0.54,1.36)	0.51	0.97 (0.59,1.59)	0.90	0.93	
Smoking status									0.12
Never	ref	0.98 (0.77,1.24)	0.84	0.91 (0.70,1.20)	0.51	1.05 (0.83,1.32)	0.69	0.47	
Former	ref	1.82 (1.08,3.05)	0.03	1.22 (0.84,1.78)	0.28	1.32 (0.88,1.98)	0.18	0.92	
Now	ref	0.81 (0.50,1.31)	0.37	0.69 (0.43,1.11)	0.12	1.10 (0.72,1.68)	0.64	0.22	
Alcohol drinking									0.73
Former	ref	0.84 (0.41,1.69)	0.61	0.62 (0.31,1.24)	0.17	0.75 (0.41,1.36)	0.33	0.54	
Heavy	ref	1.03 (0.71,1.49)	0.87	0.89 (0.58,1.35)	0.57	1.09 (0.74,1.62)	0.64	0.58	
Moderate	ref	1.10 (0.68,1.78)	0.70	0.87 (0.57,1.34)	0.53	1.07 (0.61,1.88)	0.81	0.77	
Mild	ref	1.17 (0.85,1.61)	0.33	0.96 (0.70,1.33)	0.82	0.96 (0.69,1.33)	0.81	0.52	
Never	ref	0.83 (0.47,1.47)	0.51	0.83 (0.48,1.41)	0.48	1.53 (0.89,2.62)	0.12	0.03	
PIR									0.69
<1.5	ref	0.91 (0.64,1.28)	0.57	0.90 (0.62,1.30)	0.56	1.18 (0.85,1.65)	0.31	0.1	
1.5–3.5	ref	1.14 (0.85,1.53)	0.38	0.81 (0.55,1.18)	0.27	1.11 (0.78,1.57)	0.55	0.49	
>3.5	ref	1.06 (0.73,1.52)	0.77	0.93 (0.63,1.35)	0.69	0.98 (0.74,1.31)	0.91	0.9	
Caloric intake									0.23
<1913	ref	0.96 (0.66,1.40)	0.82	1.05 (0.75,1.47)	0.77	1.09 (0.81,1.46)	0.58	0.47	
≥1913	ref	1.15 (0.88,1.51)	0.29	0.83 (0.61,1.12)	0.22	1.08 (0.81,1.43)	0.61	0.48	
Hypertension									0.33
No	ref	0.97 (0.75,1.27)	0.85	0.81 (0.62,1.06)	0.12	0.98 (0.77,1.26)	0.90	0.6	
Yes	ref	1.29 (0.80,2.07)	0.28	1.12 (0.69,1.81)	0.65	1.34 (0.98,1.82)	0.07	0.18	
Heart attack									0.18
No	ref	1.03 (0.81,1.33)	0.79	0.90 (0.71,1.14)	0.37	1.06 (0.88,1.28)	0.53	0.32	
Yes	ref	1.76 (0.47,6.51)	0.39	0.40 (0.09,1.87)	0.24	0.68 (0.25,1.84)	0.44	0.45	
Stroke									0.2
No	ref	1.04 (0.82,1.33)	0.72	0.89 (0.70,1.12)	0.31	1.08 (0.90,1.29)	0.42	0.24	
Yes	ref	0.86 (0.22,3.39)	0.82	1.28 (0.35,4.72)	0.70	0.44 (0.09,2.07)	0.29	0.17	
Coronary heart disease									0.1
No	ref	1.02 (0.80,1.32)	0.85	0.88 (0.69,1.12)	0.29	1.06 (0.87,1.27)	0.57	0.29	
Yes	ref	2.60 (0.59,11.53)	0.20	2.86 (0.47,17.52)	0.25	0.84 (0.19, 3.71)	0.81	0.25	
Diabetes									0.18
No	ref	1.04 (0.81,1.35)	0.74	0.85 (0.66,1.08)	0.18	1.05 (0.84,1.31)	0.68	0.41	
DM	ref	1.41 (0.77,2.59)	0.25	2.07 (1.09,3.94)	0.03	1.05 (0.60,1.81)	0.87	0.41	
IFG	ref	0.76 (0.35,1.65)	0.48	1.24 (0.51,3.04)	0.62	1.13 (0.37,3.49)	0.82	0.69	
IGT	ref	0.83 (0.27,2.59)	0.74	0.50 (0.14,1.82)	0.28	1.46 (0.51,4.14)	0.46	0.22	

The subgroup analyses were adjusted for all covariates except the stratification variable itself.

**Figure 3:**
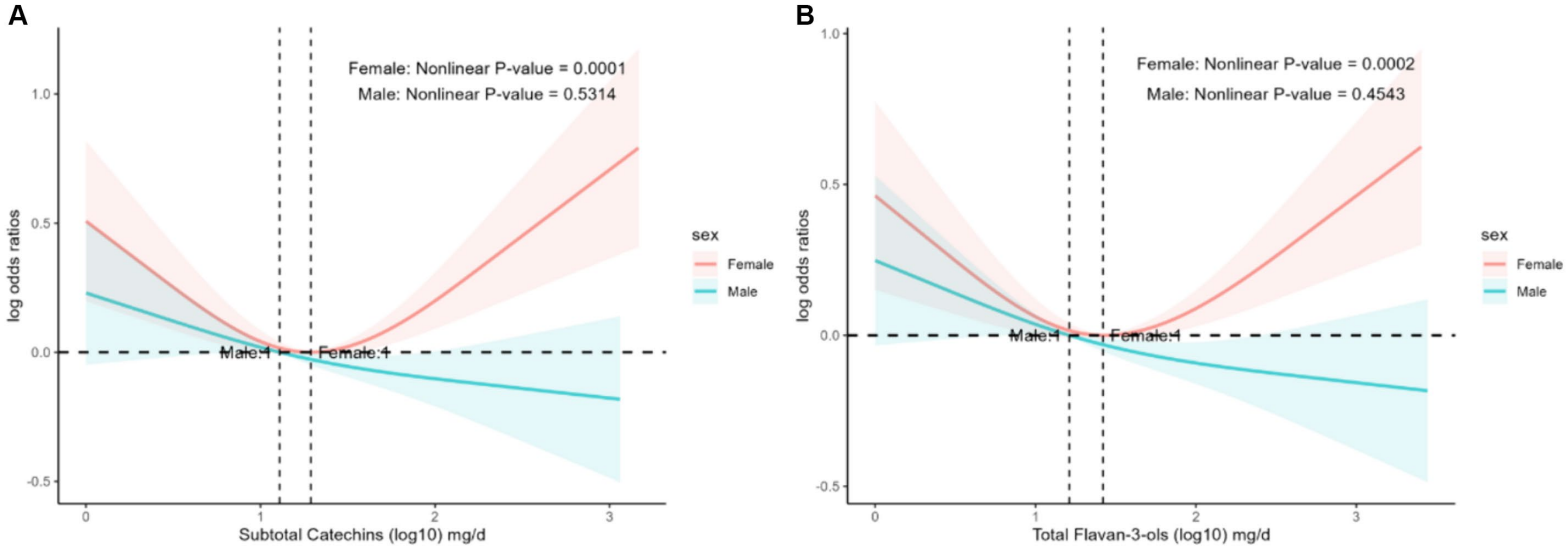
The association of flavonoid intake with prevalence of hyperlipidemia on sex by restricted cubic splines. The y-axis stands for the Log odds ratio of hyperlipidemia, and the X-axis stands for the log10 transformed intake of subtotal catechins **(A)** and total flavan-3-ols **(B)**. Models by restricted cubic splines were adjusted for age, race, caloric intake, smoking status, alcohol drinking, education, PIR, protein, total fat, total sfat, total mfat, total pfat, total cholesterol, vitamin D, vitamin E, ACR, eGFR, AST/ALT, lipid-lowering drugs, hypertension, heart attack, stroke, coronary heart disease, and diabetes.

There was an interaction between the prevalence of hyperlipidemia and flavonoid intake in sex stratification. We used restricted cubic splines analysis to evaluate the association between flavonoid intake and the prevalence of hyperlipidemia in sex stratification. The nonlinear associations between hyperlipidemia prevalence and subtotal catechins intake (Figure 3A, *p* = 0.0001) and total flavan-3-ols intake (Figure 3B, *p* = 0.0002) were significant among female participants.

## 4 Discussion

This was the first study to explore the relationship between dietary flavonoid intake and hyperlipidemia in US adults. This study analyzed the relationship between dietary flavonoid intake and hyperlipidemia using NHANES 2007–2010 and 2017–2018 data. The results demonstrated that moderate intake of dietary flavonoids can reduce the prevalence of hyperlipidemia. In model 3, we observed that compared with the first quartile, there was an inverse relationship between subtotal catechins intake and the prevalence of hyperlipidemia in the third quartile [0.74 (0.56, 0.98), *p* = 0.04]. However, the *p*-value for the trend was insignificant (*p* = 0.6). Similarly, there was an inverse relationship between total flavan-3-ols intake and the prevalence of hyperlipidemia in the third quartile [0.76 (0.59, 0.98), *p* = 0.03], with a non-significant *p*-value for trend (*p* = 0.83). Compared with the first quartile, there was an inverse relationship between total anthocyanins intake and the prevalence of hyperlipidemia in the third [0.77 (0.62, 0.95), *p* = 0.02] and fourth quartiles [0.77 (0.60, 0.98), *p* = 0.04], the p-value for trend was significant (*p* = 0.04). Total flavonols intake in the fourth quartile [0.75 (0.60, 0.94), *p* = 0.02] was inversely related to the prevalence of hyperlipidemia, and the p-value for trend was significant (*p* = 0.02). In comparison to the first quartile, we discovered that the third quartile showed an inverse connection between the prevalence of hyperlipidemia and subtotal catechin intake [0.74 (0.56, 0.98), *p* = 0.04]. There was an inverse relationship between total flavan-3-ols intake in the third quartile [0.76 (0.59, 0.98), *p* = 0.02] and the prevalence of hyperlipidemia. Total anthocyanin intake was inversely related to the prevalence of hyperlipidemia in the third quartile [0.77 (0.62, 0.95), *p* = 0.02] and the fourth quartile [0.77 (0.60, 0.98), *p* = 0.04]. Total flavonols intake in the fourth quartile [0.75 (0.60, 0.94), *p* = 0.02] was inversely related to the prevalence of hyperlipidemia. Through restricted cubic splines analysis, we found that subtotal catechins intake and total flavan-3-ols intake had a nonlinear relationship with the prevalence of hyperlipidemia.

Hyperlipidemia is a major risk factor for cardiovascular diseases. Therefore, improving hyperlipidemia is important for cardiovascular diseases. Flavonoids play a crucial role in lipid metabolism (35, 36). Green tea, black rice, blueberries, mulberries, and raspberries are rich in flavonoids. Green tea protects against hyperlipidemia (37, 38), and the catechins in green tea are essential for health promotion by reducing body weight, decreasing the accumulation of hepatic lipid droplets, preventing hepatic fat accumulation, and significantly lowering serum TC and LDL cholesterol concentrations (39–42). (−)-Epicatechin in subtotal catechins, a natural flavanol monomer found in cocoa, green tea, and various other plant foods, improved blood lipid levels in hyperlipidemic rats, reduced lipid peroxidation, inhibited pro-inflammatory cytokines, and lowered serum AST and ALT, protecting the liver from excessive fat accumulation (43). However, the low bioavailability of catechins limits their therapeutic potential. The addition of lemon juice increased plasma catechin levels significantly (44). Catechins and their derivatives epigallocatechin-3-gallate and (−) -epigallocatechin promoted cholesterol reduction by inhibiting the synthesis of hydroxy-3-methylglutaryl-CoA reductase (45).

Anthocyanins are common in the diet for their protective effects against hyperlipidemia (46). In a double-blind, randomized, placebo-controlled trial, 122 hypercholesterolemic subjects were randomized into two groups, taking either 160 mg of anthocyanins or a placebo twice daily for 24 weeks. Anthocyanin supplementation significantly increased HDL cholesterol, decreased LDL cholesterol concentrations, and increased paraoxonase 1 activity and cholesterol efflux capacity (47). Two randomized, double-blind studies showed that anthocyanins can reduce the inflammatory response in patients with hypercholesterolemia (48, 49). Dietary black rice anthocyanins may prevent obesity-associated hyperlipidemia, hepatic steatosis, and insulin resistance by influencing the gut microbiota and lipid metabolism (50). Blueberries are rich in bioactive anthocyanins with antioxidant properties. Intervention with blueberry anthocyanin extract in streptozotocin-induced diabetic mice reduced body weight, increased AMPK activity, and lowered blood and urine glucose, triglyceride, and total cholesterol levels (51). AMPK can lower blood lipids by inhibiting lipid synthesis of effectors and promoting the activity of HSL in lipolysis (52), suggesting that blueberry anthocyanins may improve hyperlipidemia by activating the AMPK signaling pathway. Mulberry anthocyanins have a hypolipidemic effect by activating AMPK phosphorylation, inhibiting lipid biosynthesis, and stimulating lipolysis (53). Raspberry anthocyanins may alleviate oxidative stress and regulate lipid metabolism (54). Total flavones include apigenin and luteolin. Apigenin lowers blood lipid levels (55), reduces lipid accumulation in adipocytes, and promotes browning of white adipocytes through autophagy inhibition, thereby ameliorating abnormalities in lipid metabolism (56). Luteolin improves lipid levels and hepatic steatosis (57, 58). T These studies have demonstrated the ability of flavonoids to regulate lipid metabolism and have a protective effect against hyperlipidemia.

When we analyzed the subgroups, we found that gender influenced the relationship between flavonoid intake and the prevalence of hyperlipidemia, with different trends in the prevalence of hyperlipidemia in women and men as flavonoid intake increased. In women, there was a statistically significant nonlinear correlation between the prevalence of hyperlipidemia and subtotal catechins intake (Figure 3A, *p* = 0.0001) and total flavan-3-ols intake (Figure 3B, *p* = 0.0002). However, the prevalence of hyperlipidemia in women showed a U-shaped curve with increasing flavonoid intake, which was different from the trend observed in men. This finding may be related to flavonoids being phytoestrogens, and there is a relationship between estrogen and lipid levels (59–61). Previous studies found that ApoC3 has a vital role in the lipoprotein lipase-mediated hydrolysis of triglyceride-rich lipoproteins, and knockdown of the ApoC3 gene significantly lowered triglyceride levels and elevated HDL cholesterol levels in hypertriglyceridemic patients (62). Estrogen inhibits ApoC3 expression, thereby reducing triglyceride levels (63). Unfortunately, no large-scale clinical studies are exploring the effect of sex on the prevalence of hyperlipidemia and flavonoids, and it is expected that large-scale prospective studies will be conducted in the future.

This study showed that among participants with hyperlipidemia, 57.28% were over 60 years old, and the average age of participants with hyperlipidemia was older than that of participants without hyperlipidemia, [48.90(0.37) vs. 38.89(0.51), *p* < 0.0001]. Among participants with hyperlipidemia, non-Hispanic White and Mexican Americans accounted for the largest proportions, 70.98, and 8.56%, respectively. However, in subgroup analysis, different races and age groups did not affect the relationship between flavonoids and hyperlipidemia prevalence (Table 3; Supplementary Tables 3–5, *p* for interaction >0.05).

The results of this study showed that among participants with hyperlipidemia, the proportion of former drinkers and moderate drinkers was large, accounting for 12.46 and 39.31%, respectively. The proportion of current smokers and former smokers is large, 19.25 and 24.94%, respectively. However, in subgroup analysis, alcohol drinking and smoking status had no significant impact on the relationship between the prevalence of hyperlipidemia and anthocyanin intake (Table 3; Supplementary Tables 3–5, *p* for interaction >0.05). It has been reported that alcohol consumption can increase plasma triglyceride levels and cause abnormalities in lipid metabolism, leading to hyperlipidemia (64–66). Studies have found that smoking increases total blood cholesterol levels and lowers beneficial high-density lipoprotein (HDL) levels (67, 68). Therefore, smoking cessation and moderate alcohol consumption are crucial in the management of hyperlipidemia.

Our study had several strengths. First, to our knowledge, this was the first study to explore the relationship between dietary flavonoid intake and hyperlipidemia in US adults. Second, we explored the nonlinear relationship between dietary flavonoid intake and hyperlipidemia compared with previous studies. Third, our study showed consistent results from curve fitting and piecewise linear regression, indicating that the results were stable and reliable. Finally, we conducted a subgroup analysis and found that dietary flavonoid intake trends and hyperlipidemia prevalence were different in different genders. However, our study also had several limitations. This study was a cross-sectional study that cannot draw causal inferences. Dietary flavonoid intake was calculated based on 24-h dietary recall, which may be subject to recall bias. We look forward to future prospective studies with large samples and different genders.

## 5 Conclusion

Our study demonstrated that specific intakes of flavonoids were inversely associated with the risk of hyperlipidemia. We observed an inverse association between the risk of hyperlipidemia and moderate intake of subtotal catechins, flavan-3-ols, total anthocyanins, and total flavones. Our findings may provide valuable information for customized nutritional interventions to manage hyperlipidemia.

## Data availability statement

Publicly available datasets were analyzed in this study. This data can be found at: All NHANES data for this study are publicly available and can be found at: https://wwwn.cdc.gov/nchs/nhanes.

## Ethics statement

The studies involving humans were approved by Institutional Review Board Statement: The study was conducted in accordance with the Declaration of Helsinki and was approved by the Institutional Review Board of the National Center for Health Statistics (protocol #2005–06, #2011–17, #2018–01). The studies were conducted in accordance with the local legislation and institutional requirements. The participants provided their written informed consent to participate in this study.

## Author contributions

YW: Writing – review & editing, Writing – original draft, Validation, Project administration, Methodology, Conceptualization. DM: Writing – original draft, Formal analysis, Data curation. LY: Writing – review & editing, Data curation. WT: Writing – review & editing, Investigation. TW: Writing – review & editing, Software. XC: Writing – review & editing, Visualization. QS: Writing – review & editing, Supervision, Project administration. HX: Writing – review & editing, Supervision, Resources, Funding acquisition.

## Footnotes

^1^ https://www.cdc.gov/nchs/nhanes/index.htm
