# Supplementary material for: The associations between dietary flavonoid intake and hyperlipidemia: data from the national health and nutrition examination survey 2007–2010 and 2017–2018
Source: Front Nutr. 2024 May 31;11:1374970. doi: 10.3389/fnut.2024.1374970 (PMC11176614; doi:10.3389/fnut.2024.1374970)
Supplement: Supplementary file 1 [file Table_1.DOCX]

Supplementary Table 1 Characteristics of participants according to whether they take lipid-lowering drugs

| Variable | Participants without taking lipid-lowering drugs | Participants with taking lipid-lowering drugs | *P-*value |
| --- | --- | --- | --- |
| N | 7187 | 1753 |  |
| Age (years) | 42.92(0.35) | 61.53(0.37) | < 0.0001 |
| Sex, N (%) |  |  | 0.1 |
| Female | 3798(53.09) | 840(49.68) |  |
| Male | 3389(46.91) | 913(50.32) |  |
| Race, N (%) |  |  | < 0.0001 |
| Non-Hispanic White | 3185(67.79) | 938(77.13) |  |
| Non-Hispanic Black | 1417(10.57) | 308(8.48) |  |
| Mexican American | 1270(9.04) | 223(4.96) |  |
| Others | 1315(12.60) | 284(9.43) |  |
| Education, N (%) |  |  | 0.01 |
| Less than high school | 1640(14.00) | 465(15.57) |  |
| High school or equivalent | 1654(24.42) | 433(28.94) |  |
| Some college or AA degree | 2232(31.88) | 465(27.14) |  |
| College graduate or above | 1661(29.70) | 390(28.35) |  |
| Smoking status, N (%) |  |  | < 0.0001 |
| Now | 1561(20.05) | 248(12.96) |  |
| Former | 1476(21.17) | 664(36.48) |  |
| Never | 4150(58.78) | 841(50.56) |  |
| Alcohol drinking, N (%) |  |  | < 0.0001 |
| Former | 897(9.67) | 377(16.92) |  |
| Heavy | 1756(25.06) | 167(9.42) |  |
| Mild | 2378(36.62) | 742(47.24) |  |
| Moderate | 1299(19.19) | 204(16.01) |  |
| Never | 857(9.46) | 263(10.42) |  |
| PIR, N (%) |  |  | 0.001 |
| <1.5 | 2619(24.96) | 517(18.89) |  |
| 1.5-3.5 | 2332(30.33) | 600(29.31) |  |
| >3.5 | 2236(44.70) | 636(51.80) |  |
| BMI (kg/m^2^) | 28.84(0.14) | 30.71(0.19) | < 0.0001 |
| Caloric intake (kcal) | 2088.51(13.59) | 1976.90(29.16) | < 0.001 |
| Protein (g) | 82.18(0.67) | 79.78(1.27) | 0.09 |
| Carbohydrate (g) | 248.90(1.58) | 230.83(3.00) | < 0.0001 |
| Total fat (g) | 80.21(0.76) | 78.98(1.58) | 0.51 |
| Total sfat (g) | 26.29(0.28) | 25.55(0.50) | 0.22 |
| Total mfat (g) | 28.51(0.28) | 28.48(0.60) | 0.97 |
| Total pfat (g) | 18.00(0.19) | 17.80(0.43) | 0.68 |
| Total cholesterol (mg) | 286.13(3.56) | 285.01(6.97) | 0.88 |
| Vitamin D (mcg) | 4.51(0.07) | 4.79(0.15) | 0.11 |
| Vitamin E (mg) | 8.21(0.13) | 8.24(0.22) | 0.93 |
| ACR (mg/g) | 24.08(2.48) | 45.59(10.43) | 0.06 |
| eGFR (ml/min/1.73m^2^) | 98.72(0.54) | 80.70(0.70) | < 0.0001 |
| AST/ALT | 1.11(0.01) | 1.08(0.01) | 0.09 |
| Hyperlipidemia, N (%) |  |  | < 0.0001 |
| No | 2420(34.44) | 0(0.00) |  |
| Yes | 4767(65.56) | 1753(100.00) |  |
| Hypertension, N (%) |  |  | < 0.0001 |
| No | 4976(73.18) | 490(32.04) |  |
| Yes | 2211(26.82) | 1263(67.96) |  |
| Stroke |  |  | < 0.0001 |
| No | 7040(98.49) | 1584(91.74) |  |
| Yes | 147(1.51) | 169(8.26) |  |
| Heart attack, N (%) |  |  | < 0.0001 |
| No | 7064(98.75) | 1543(89.70) |  |
| Yes | 123(1.25) | 210(10.30) |  |
| Coronary heart disease, N (%) |  |  | < 0.0001 |
| No | 7115(99.21) | 1499(85.83) |  |
| Yes | 72(0.79) | 254(14.17) |  |
| Diabetes, N (%) |  |  | < 0.0001 |
| No | 5775(84.81) | 809(53.38) |  |
| DM | 797(7.94) | 781(36.42) |  |
| IFG | 361(4.31) | 106(7.18) |  |
| IGT | 254(2.94) | 57(3.03) |  |
| Dietary intake of flavonoids (mg/day) | |  |  |
| Subtotal Catechins | 77.12(4.70) | 81.55(4.95) | 0.48 |
| Total Isoflavones | 2.33(0.22) | 1.35(0.20) | 0.002 |
| Total Anthocyanidins | 13.92(0.93) | 16.66(1.73) | 0.12 |
| Total Flavan-3-ols | 173.22(8.40) | 183.24(11.67) | 0.43 |
| Total Flavanones | 12.22(0.44) | 12.95(0.73) | 0.37 |
| Total Flavones | 0.90(0.03) | 0.99(0.06) | 0.18 |
| Total Flavonols | 18.75(0.37) | 18.73(0.59) | 0.97 |
| Total Sum of all 29 flavonoids | 221.35(8.68) | 233.90(12.02) | 0.32 |

Supplementary table 2 Characteristics of participants’ flavonoid dietary assessment

| Variable | Participants without hyperlipidemia | Participants with hyperlipidemia | *P-*value |
| --- | --- | --- | --- |
| N | 2420 | 6520 |  |
| Dietary intake of flavonoids (mg/day) | |  |  |
| Total Isoflavones | 3.23(0.36) | 1.74(0.22) | 0.001 |
| Daidzein | 1.18(0.13) | 0.67(0.08) | 0.002 |
| Genistein | 1.78(0.20) | 0.94(0.12) | < 0.001 |
| Glycitein | 0.26(0.03) | 0.14(0.02) | 0.001 |
| Total Anthocyanidins | 14.46(1.42) | 14.35(0.98) | 0.94 |
| Cyanidin | 2.30(0.28) | 2.74(0.24) | 0.22 |
| Petunidin | 1.27(0.20) | 1.21(0.15) | 0.8 |
| Delphinidin | 1.84(0.30) | 1.70(0.19) | 0.64 |
| Malvidin | 5.05(0.57) | 5.03(0.42) | 0.97 |
| Pelargonidin | 1.66(0.19) | 1.53(0.12) | 0.5 |
| Peonidin | 2.34(0.26) | 2.13(0.27) | 0.6 |
| Total Flavanones | 12.69(0.65) | 12.20(0.40) | 0.43 |
| Eriodictyol | 0.18(0.01) | 0.18(0.02) | 0.71 |
| Hesperetin | 9.20(0.49) | 8.74(0.29) | 0.34 |
| Naringenin | 3.31(0.20) | 3.28(0.15) | 0.86 |
| Total Flavones | 0.95(0.04) | 0.90(0.03) | 0.33 |
| Apigenin | 0.21(0.02) | 0.20(0.01) | 0.83 |
| Luteolin | 0.74(0.03) | 0.70(0.02) | 0.25 |
| Total Flavonols | 18.36(0.36) | 18.90(0.42) | 0.21 |
| Isorhamnetin | 0.91(0.03) | 0.86(0.02) | 0.16 |
| Kaempferol | 4.88(0.15) | 4.75(0.13) | 0.39 |
| Myricetin | 1.43(0.07) | 1.62(0.06) | 0.01 |
| Quercetin | 11.13(0.22) | 11.67(0.24) | 0.05 |
| Total Flavan-3-ols | 151.81(8.45) | 184.18(9.27) | 0.002 |
| Catechin | 7.78(0.27) | 7.90(0.22) | 0.67 |
| Epigallocatechin | 14.46(0.91) | 18.25(1.27) | 0.01 |
| Epicatechin | 9.63(0.33) | 10.39(0.34) | 0.09 |
| Epicatechin-3-gallate | 9.28(0.60) | 11.76(0.80) | 0.01 |
| Epigallocatechin-3-gallate | 24.63(1.85) | 31.98(2.77) | 0.02 |
| Theaflavin | 1.38(0.09) | 1.67(0.10) | 0.01 |
| Thearubigins | 79.20(5.09) | 95.70(5.18) | 0.01 |
| Theaflavin-3,3’-digallate | 1.53(0.10) | 1.84(0.11) | 0.01 |
| Theaflavin-3’-gallate | 1.30(0.09) | 1.56(0.09) | 0.02 |
| Theaflavin 3-gallate | 1.09(0.07) | 1.32(0.08) | 0.01 |
| Gallocatechin | 1.53(0.09) | 1.83(0.10) | 0.01 |
| Subtotal Catechins | 67.31(3.83) | 82.10(5.37) | 0.02 |
| Total Sum of all 29 flavonoids | 201.49(8.99) | 232.29(9.57) | 0.004 |

Supplementary Table 3 Subgroup analysis between hyperlipidemia and total flavan-3-ols

| Variables | Q1 | Q2 |  | Q3 |  | Q4 |  |  |  |
| --- | --- | --- | --- | --- | --- | --- | --- | --- | --- |
|  |  | OR (95%CI) | p  Value | OR (95%CI) | p  Value | OR (95%CI) | p  Value | p for trend | p for interaction |
| Age |  |  |  |  |  |  |  |  | 0.96 |
| 20-39 | ref | 0.92(0.67,1.27) | 0.60 | 0.81(0.56,1.16) | 0.25 | 0.96(0.69,1.35) | 0.81 | 0.76 |  |
| 40-59 | ref | 0.94(0.62,1.42) | 0.75 | 0.72(0.50,1.04) | 0.08 | 0.83(0.61,1.13) | 0.24 | 0.66 |  |
| ≥60 | ref | 0.84(0.50,1.41) | 0.50 | 0.77(0.42,1.43) | 0.40 | 0.72(0.40,1.31) | 0.28 | 0.4 |  |
| Race |  |  |  |  |  |  |  |  | 0.75 |
| Non-Hispanic White | ref | 0.92(0.65,1.32) | 0.66 | 0.86(0.64,1.16) | 0.32 | 0.95(0.67,1.33) | 0.75 | 0.87 |  |
| Non-Hispanic Black | ref | 1.23(0.82,1.86) | 0.31 | 1.18(0.82,1.70) | 0.37 | 1.39(1.04,1.85) | 0.03 | 0.06 |  |
| Mexican American | ref | 0.90(0.56,1.46) | 0.67 | 0.71(0.39,1.29) | 0.25 | 0.72(0.43,1.21) | 0.21 | 0.35 |  |
| Others | ref | 1.30(0.73,2.32) | 0.36 | 1.15(0.68,1.94) | 0.60 | 1.10(0.67,1.82) | 0.69 | 0.88 |  |
| Sex |  |  |  |  |  |  |  |  | 0.01 |
| Female | ref | 0.80(0.56,1.12) | 0.18 | 0.93(0.71,1.23) | 0.62 | 1.11(0.85,1.45) | 0.44 | 0.05 |  |
| Male | ref | 1.31(0.97,1.75) | 0.07 | 0.91(0.66,1.24) | 0.53 | 0.92(0.68,1.24) | 0.58 | 0.23 |  |
| Education |  |  |  |  |  |  |  |  | 0.27 |
| Less than high school | ref | 0.86(0.55,1.35) | 0.49 | 1.24(0.79,1.94) | 0.34 | 0.99(0.68,1.43) | 0.94 | 0.97 |  |
| High school or equivalent | ref | 0.87(0.52,1.43) | 0.57 | 0.62(0.37,1.04) | 0.07 | 1.02(0.65,1.62) | 0.92 | 0.33 |  |
| Some college or AA degree | ref | 1.32(0.93,1.85) | 0.11 | 1.29(0.93,1.77) | 0.12 | 1.31(0.90,1.90) | 0.15 | 0.51 |  |
| College graduate or above | ref | 0.99(0.61,1.60) | 0.96 | 0.87(0.56,1.35) | 0.53 | 0.93(0.56,1.53) | 0.76 | 0.92 |  |
| Smoking status |  |  |  |  |  |  |  |  | 0.06 |
| Never | ref | 0.94(0.74,1.20) | 0.62 | 0.98(0.77,1.25) | 0.86 | 1.02(0.79,1.32) | 0.89 | 0.68 |  |
| Former | ref | 1.77(1.06,2.96) | 0.03 | 1.35(0.91,2.01) | 0.13 | 1.25(0.83,1.88) | 0.27 | 0.7 |  |
| Now | ref | 0.80(0.50,1.27) | 0.34 | 0.60(0.36,1.00) | 0.05 | 1.10(0.75,1.61) | 0.62 | 0.12 |  |
| Alcohol drinking |  |  |  |  |  |  |  |  | 0.95 |
| Former | ref | 0.82(0.41,1.64) | 0.56 | 0.65(0.32,1.30) | 0.21 | 0.72(0.40,1.30) | 0.26 | 0.46 |  |
| Heavy | ref | 0.98(0.68,1.41) | 0.91 | 0.91(0.59,1.42) | 0.68 | 1.06(0.71,1.56) | 0.78 | 0.65 |  |
| Moderate | ref | 1.14(0.69,1.88) | 0.61 | 0.84(0.55,1.31) | 0.44 | 1.01(0.58,1.76) | 0.97 | 0.94 |  |
| Mild | ref | 1.12(0.79,1.58) | 0.52 | 1.01(0.76,1.35) | 0.95 | 0.98(0.71,1.36) | 0.92 | 0.65 |  |
| Never | ref | 0.86(0.48,1.54) | 0.60 | 1.00(0.57,1.75) | 0.99 | 1.22(0.71,2.09) | 0.46 | 0.34 |  |
| PIR |  |  |  |  |  |  |  |  | 0.99 |
| <1.5 | ref | 0.92(0.65,1.30) | 0.63 | 0.96(0.66,1.40) | 0.83 | 1.02(0.74,1.41) | 0.88 | 0.63 |  |
| 1.5-3.5 | ref | 1.10(0.79,1.52) | 0.56 | 0.92(0.65,1.31) | 0.64 | 1.03(0.73,1.44) | 0.87 | 0.92 |  |
| >3.5 | ref | 1.01(0.70,1.45) | 0.95 | 0.91(0.66,1.25) | 0.55 | 1.01(0.72,1.43) | 0.94 | 0.73 |  |
| Caloric intake |  |  |  |  |  |  |  |  | 0.17 |
| <1913 | ref | 0.94(0.65,1.34) | 0.71 | 1.14(0.80,1.62) | 0.45 | 1.04(0.77,1.40) | 0.78 | 0.8 |  |
| ≥1913 | ref | 1.12(0.86,1.45) | 0.40 | 0.84(0.63,1.13) | 0.25 | 1.03(0.76,1.41) | 0.83 | 0.63 |  |
| Hypertension |  |  |  |  |  |  |  |  | 0.24 |
| No | ref | 0.94(0.72,1.22) | 0.62 | 0.85(0.66,1.10) | 0.22 | 0.94(0.73,1.21) | 0.61 | 0.94 |  |
| Yes | ref | 1.29(0.82,2.04) | 0.26 | 1.12(0.77,1.64) | 0.54 | 1.38(0.93,2.05) | 0.11 | 0.19 |  |
| Heart attack |  |  |  |  |  |  |  |  | 0.02 |
| No | ref | 1.01(0.79,1.29) | 0.96 | 0.94(0.76,1.16) | 0.56 | 1.02(0.83,1.26) | 0.84 | 0.63 |  |
| Yes | ref | 2.43(0.75,7.84) | 0.13 | 0.33(0.08,1.35) | 0.12 | 0.79(0.29,2.12) | 0.63 | 0.9 |  |
| Stroke |  |  |  |  |  |  |  |  | 0.19 |
| No | ref | 1.02(0.80,1.29) | 0.89 | 0.92(0.76,1.13) | 0.43 | 1.04(0.85,1.27) | 0.70 | 0.48 |  |
| Yes | ref | 0.87(0.23,3.31) | 0.83 | 1.24(0.33,4.65) | 0.74 | 0.42(0.09,1.99) | 0.27 | 0.14 |  |
| Coronary heart disease |  |  |  |  |  |  |  |  | 0.23 |
| No | ref | 1.00(0.78,1.28) | 0.98 | 0.92(0.75,1.13) | 0.42 | 1.02(0.83,1.25) | 0.87 | 0.59 |  |
| Yes | ref | 2.55(0.57,11.39) | 0.21 | 2.78(0.46,16.88) | 0.26 | 0.87(0.20, 3.87) | 0.85 | 0.26 |  |
| Diabetes |  |  |  |  |  |  |  |  | 0.11 |
| No | ref | 1.01(0.78,1.31) | 0.92 | 0.90(0.72,1.13) | 0.36 | 0.99(0.79,1.25) | 0.96 | 0.82 |  |
| DM | ref | 1.41(0.78,2.56) | 0.25 | 2.13(1.16,3.90) | 0.02 | 1.07(0.62,1.82) | 0.81 | 0.34 |  |
| IFG | ref | 0.85(0.42,1.71) | 0.64 | 0.71(0.27,1.87) | 0.48 | 2.32(0.68,7.86) | 0.17 | 0.08 |  |
| IGT | ref | 0.77(0.25,2.35) | 0.63 | 0.50(0.14,1.79) | 0.28 | 1.20(0.42,3.45) | 0.73 | 0.32 |  |

The subgroup analyses were adjusted for all covariates except the stratification variable itself.

Supplementary Table 4 Subgroup analysis between hyperlipidemia and total anthocyanidins

| Variables | Q1 | Q2 |  | Q3 |  | Q4 |  |  |  |
| --- | --- | --- | --- | --- | --- | --- | --- | --- | --- |
|  |  | OR (95%CI) | p  Value | OR (95%CI) | p  Value | OR (95%CI) | p  Value | p for trend | p for interaction |
| Age |  |  |  |  |  |  |  |  | 0.65 |
| 20-39 | ref | 1.23(0.90,1.69) | 0.19 | 0.86(0.65,1.15) | 0.30 | 0.81(0.59,1.10) | 0.17 | 0.06 |  |
| 40-59 | ref | 1.02(0.71,1.48) | 0.90 | 0.76(0.49,1.17) | 0.20 | 0.73(0.48,1.11) | 0.13 | 0.11 |  |
| ≥60 | ref | 1.04(0.64,1.67) | 0.88 | 0.89(0.59,1.35) | 0.58 | 1.09(0.61,1.95) | 0.77 | 0.66 |  |
| Race |  |  |  |  |  |  |  |  | 0.4 |
| Non-Hispanic White | ref | 1.19(0.88,1.62) | 0.25 | 0.93(0.70,1.23) | 0.59 | 0.96(0.73,1.27) | 0.77 | 0.43 |  |
| Non-Hispanic Black | ref | 1.25(0.86,1.82) | 0.23 | 1.01(0.67,1.52) | 0.96 | 1.04(0.71,1.54) | 0.83 | 0.84 |  |
| Mexican American | ref | 0.79(0.47,1.32) | 0.36 | 0.68(0.41,1.11) | 0.12 | 0.79(0.43,1.46) | 0.44 | 0.85 |  |
| Others | ref | 1.69(0.97,2.95) | 0.07 | 1.12(0.67,1.90) | 0.66 | 1.68(1.04,2.70) | 0.04 | 0.12 |  |
| Sex |  |  |  |  |  |  |  |  | 0.1 |
| Female | ref | 0.99(0.69,1.40) | 0.93 | 0.92(0.68,1.26) | 0.60 | 0.86(0.66,1.13) | 0.28 | 0.26 |  |
| Male | ref | 1.45(1.09,1.92) | 0.01 | 0.92(0.71,1.19) | 0.50 | 1.26(0.91,1.76) | 0.16 | 0.39 |  |
| Education |  |  |  |  |  |  |  |  | 0.19 |
| Less than high school | ref | 0.97(0.61,1.55) | 0.90 | 1.13(0.79,1.63) | 0.50 | 1.07(0.64,1.78) | 0.79 | 0.76 |  |
| High school or equivalent | ref | 0.98(0.63,1.54) | 0.94 | 0.72(0.45,1.13) | 0.15 | 1.00(0.64,1.56) | 0.99 | 0.81 |  |
| Some college or AA degree | ref | 1.42(0.97,2.10) | 0.07 | 1.17(0.88,1.55) | 0.27 | 1.59(1.09,2.32) | 0.02 | 0.03 |  |
| College graduate or above | ref | 1.41(0.87,2.29) | 0.16 | 0.90(0.58,1.42) | 0.65 | 0.98(0.67,1.43) | 0.90 | 0.44 |  |
| Smoking status |  |  |  |  |  |  |  |  | 0.09 |
| Never | ref | 1.18(0.85,1.64) | 0.32 | 1.00(0.73,1.36) | 0.99 | 0.95(0.73,1.24) | 0.70 | 0.3 |  |
| Former | ref | 1.40(0.90,2.17) | 0.13 | 0.79(0.52,1.21) | 0.28 | 1.44(0.97,2.14) | 0.07 | 0.05 |  |
| Now | ref | 0.91(0.60,1.36) | 0.62 | 0.92(0.49,1.70) | 0.78 | 0.61(0.36,1.02) | 0.06 | 0.05 |  |
| Alcohol drinking |  |  |  |  |  |  |  |  | 0.32 |
| Former | ref | 0.59(0.32,1.12) | 0.10 | 0.62(0.35,1.10) | 0.10 | 0.76(0.38,1.51) | 0.42 | 0.99 |  |
| Heavy | ref | 1.22(0.88,1.70) | 0.23 | 0.86(0.55,1.35) | 0.51 | 0.75(0.46,1.23) | 0.25 | 0.13 |  |
| Moderate | ref | 1.10(0.60,2.04) | 0.75 | 1.04(0.60,1.78) | 0.89 | 1.02(0.69,1.52) | 0.91 | 0.91 |  |
| Mild | ref | 1.61(1.08,2.39) | 0.02 | 1.10(0.83,1.45) | 0.49 | 1.35(0.97,1.88) | 0.07 | 0.47 |  |
| Never | ref | 0.92(0.51,1.66) | 0.78 | 0.72(0.41,1.27) | 0.25 | 1.12(0.63,1.99) | 0.69 | 0.33 |  |
| PIR |  |  |  |  |  |  |  |  | 0.76 |
| <1.5 | ref | 0.96(0.70,1.32) | 0.79 | 0.81(0.58,1.13) | 0.21 | 0.91(0.64,1.28) | 0.57 | 0.68 |  |
| 1.5-3.5 | ref | 1.24(0.90,1.71) | 0.18 | 0.92(0.67,1.27) | 0.61 | 1.12(0.79,1.60) | 0.52 | 0.76 |  |
| >3.5 | ref | 1.42(0.91,2.22) | 0.12 | 1.04(0.72,1.52) | 0.82 | 1.11(0.77,1.61) | 0.56 | 0.88 |  |
| Caloric intake |  |  |  |  |  |  |  |  | 0.26 |
| <1913 | ref | 1.08(0.79,1.49) | 0.63 | 0.91(0.71,1.18) | 0.49 | 1.16(0.86,1.57) | 0.32 | 0.3 |  |
| ≥1913 | ref | 1.36(1.05,1.76) | 0.02 | 0.97(0.75,1.27) | 0.84 | 1.00(0.75,1.35) | 0.98 | 0.43 |  |
| Hypertension |  |  |  |  |  |  |  |  | 0.06 |
| No | ref | 1.24(0.94,1.64) | 0.13 | 0.93(0.74,1.19) | 0.57 | 0.96(0.74,1.24) | 0.76 | 0.34 |  |
| Yes | ref | 1.03(0.71,1.48) | 0.88 | 0.85(0.54,1.33) | 0.47 | 1.43(0.93,2.21) | 0.10 | 0.03 |  |
| Heart attack |  |  |  |  |  |  |  |  | 0.17 |
| No | ref | 1.23(0.98,1.55) | 0.08 | 0.95(0.79,1.14) | 0.59 | 1.07(0.86,1.33) | 0.52 | 0.93 |  |
| Yes | ref | 0.27(0.09,0.85) | 0.03 | 0.22(0.07,0.72) | 0.01 | 0.23(0.05,0.95) | 0.04 | 0.33 |  |
| Stroke |  |  |  |  |  |  |  |  | 0.06 |
| No | ref | 1.23(0.98,1.55) | 0.07 | 0.94(0.78,1.13) | 0.51 | 1.06(0.85,1.31) | 0.60 | 0.97 |  |
| Yes | ref | 0.31(0.10,0.96) | 0.04 | 0.35(0.09,1.32) | 0.12 | 0.80(0.22,2.91) | 0.73 | 0.26 |  |
| Coronary heart disease |  |  |  |  |  |  |  |  | 0.41 |
| No | ref | 1.21(0.97,1.52) | 0.09 | 0.94(0.78,1.13) | 0.50 | 1.04(0.84,1.29) | 0.71 | 0.87 |  |
| Yes | ref | 0.80(0.15,4.14) | 0.78 | 0.45(0.10,1.95) | 0.28 | 1.51(0.34,6.67) | 0.58 | 0.28 |  |
| Diabetes |  |  |  |  |  |  |  |  | 0.2 |
| No | ref | 1.21(0.93,1.58) | 0.16 | 0.90(0.74,1.09) | 0.27 | 1.01(0.82,1.25) | 0.92 | 0.69 |  |
| DM | ref | 0.78(0.39,1.57) | 0.48 | 1.06(0.56,2.01) | 0.85 | 1.18(0.61,2.28) | 0.63 | 0.31 |  |
| IFG | ref | 1.84(0.80,4.22) | 0.14 | 1.61(0.55,4.72) | 0.38 | 2.69(1.09,6.66) | 0.03 | 0.07 |  |
| IGT | ref | 0.60(0.15,2.35) | 0.45 | 0.22(0.05,0.96) | 0.04 | 0.31(0.06,1.60) | 0.15 | 0.47 |  |

The subgroup analyses were adjusted for all covariates except the stratification variable itself.

Supplementary Table 5 Subgroup analysis between hyperlipidemia and Total Flavonols

| Variables | Q1 | Q2 |  | Q3 |  | Q4 |  |  |  |
| --- | --- | --- | --- | --- | --- | --- | --- | --- | --- |
|  |  | OR (95%CI) | p  Value | OR (95%CI) | p  Value | OR (95%CI) | p  Value | p for trend | p for interaction |
| Age |  |  |  |  |  |  |  |  | 0.75 |
| 20-39 | ref | 0.73(0.57,0.95) | 0.02 | 0.80(0.62,1.03) | 0.08 | 0.79(0.57,1.10) | 0.16 | 0.38 |  |
| 40-59 | ref | 0.95(0.67,1.34) | 0.74 | 1.04(0.74,1.47) | 0.82 | 0.83(0.60,1.15) | 0.26 | 0.26 |  |
| ≥60 | ref | 0.77(0.48,1.23) | 0.27 | 0.65(0.38,1.10) | 0.10 | 0.63(0.36,1.13) | 0.12 | 0.16 |  |
| Race |  |  |  |  |  |  |  |  | 0.73 |
| Non-Hispanic White | ref | 0.82(0.64,1.06) | 0.13 | 0.86(0.66,1.11) | 0.23 | 0.78(0.62,0.98) | 0.04 | 0.1 |  |
| Non-Hispanic Black | ref | 0.94(0.68,1.32) | 0.73 | 1.10(0.83,1.47) | 0.49 | 1.23(0.94,1.62) | 0.13 | 0.06 |  |
| Mexican American | ref | 0.79(0.54,1.17) | 0.23 | 0.76(0.46,1.25) | 0.27 | 0.63(0.40,0.99) | 0.05 | 0.07 |  |
| Others | ref | 0.93(0.59,1.47) | 0.75 | 0.92(0.62,1.37) | 0.68 | 0.97(0.57,1.68) | 0.93 | 0.98 |  |
| Sex |  |  |  |  |  |  |  |  | 0.06 |
| Female | ref | 0.92(0.72,1.17) | 0.49 | 0.96(0.73,1.25) | 0.74 | 1.14(0.84,1.55) | 0.39 | 0.26 |  |
| Male | ref | 0.78(0.55,1.11) | 0.16 | 0.84(0.61,1.15) | 0.26 | 0.68(0.49,0.94) | 0.02 | 0.02 |  |
| Education |  |  |  |  |  |  |  |  | 0.83 |
| Less than high school | ref | 0.96(0.66,1.40) | 0.83 | 1.20(0.79,1.83) | 0.38 | 1.02(0.63,1.63) | 0.95 | 0.82 |  |
| High school or equivalent | ref | 0.65(0.45,0.95) | 0.03 | 0.77(0.53,1.12) | 0.17 | 0.72(0.44,1.19) | 0.20 | 0.42 |  |
| Some college or AA degree | ref | 1.08(0.78,1.48) | 0.63 | 1.06(0.78,1.44) | 0.70 | 1.04(0.73,1.47) | 0.83 | 0.95 |  |
| College graduate or above | ref | 0.88(0.57,1.36) | 0.57 | 0.93(0.60,1.45) | 0.76 | 0.95(0.61,1.48) | 0.81 | 0.95 |  |
| Smoking status |  |  |  |  |  |  |  |  | 0.62 |
| Never | ref | 0.80(0.64,0.99) | 0.04 | 0.95(0.74,1.22) | 0.67 | 0.89(0.71,1.12) | 0.31 | 0.82 |  |
| Former | ref | 1.15(0.81,1.64) | 0.42 | 0.91(0.61,1.37) | 0.65 | 0.97(0.68,1.40) | 0.88 | 0.62 |  |
| Now | ref | 0.90(0.59,1.38) | 0.63 | 0.89(0.58,1.36) | 0.57 | 0.79(0.50,1.27) | 0.33 | 0.34 |  |
| Alcohol drinking |  |  |  |  |  |  |  |  | 0.06 |
| Former | ref | 0.71(0.39,1.29) | 0.24 | 0.61(0.35,1.06) | 0.08 | 0.48(0.27,0.84) | 0.01 | 0.02 |  |
| Heavy | ref | 1.11(0.79,1.57) | 0.55 | 1.19(0.79,1.78) | 0.40 | 1.28(0.91,1.80) | 0.15 | 0.21 |  |
| Moderate | ref | 0.67(0.39,1.17) | 0.15 | 0.56(0.37,0.84) | 0.01 | 0.74(0.47,1.16) | 0.18 | 0.42 |  |
| Mild | ref | 0.90(0.62,1.31) | 0.58 | 1.02(0.76,1.36) | 0.91 | 0.78(0.56,1.09) | 0.14 | 0.12 |  |
| Never | ref | 0.85(0.46,1.58) | 0.61 | 1.47(0.76,2.87) | 0.25 | 1.52(0.76,3.05) | 0.23 | 0.1 |  |
| PIR |  |  |  |  |  |  |  |  | 0.15 |
| <1.5 | ref | 0.82(0.61,1.12) | 0.21 | 1.10(0.78,1.53) | 0.59 | 0.91(0.66,1.27) | 0.59 | 0.96 |  |
| 1.5-3.5 | ref | 0.92(0.67,1.26) | 0.59 | 0.85(0.64,1.14) | 0.28 | 0.66(0.47,0.93) | 0.02 | 0.02 |  |
| >3.5 | ref | 0.85(0.60,1.19) | 0.33 | 0.87(0.62,1.21) | 0.40 | 1.00(0.74,1.36) | 0.99 | 0.49 |  |
| Caloric intake |  |  |  |  |  |  |  |  | 0.55 |
| <1913 | ref | 0.99(0.76,1.28) | 0.93 | 0.95(0.71,1.27) | 0.71 | 1.02(0.76,1.36) | 0.90 | 0.93 |  |
| ≥1913 | ref | 0.74(0.54,1.03) | 0.08 | 0.87(0.61,1.25) | 0.44 | 0.79(0.56,1.12) | 0.18 | 0.5 |  |
| Hypertension |  |  |  |  |  |  |  |  | 0.64 |
| No | ref | 0.83(0.66,1.06) | 0.13 | 0.94(0.75,1.19) | 0.62 | 0.84(0.68,1.02) | 0.08 | 0.18 |  |
| Yes | ref | 0.94(0.63,1.39) | 0.74 | 0.83(0.58,1.19) | 0.31 | 0.96(0.63,1.44) | 0.82 | 0.92 |  |
| Heart attack |  |  |  |  |  |  |  |  | 0.23 |
| No | ref | 0.85(0.70,1.03) | 0.09 | 0.91(0.75,1.10) | 0.30 | 0.87(0.71,1.05) | 0.14 | 0.31 |  |
| Yes | ref | 2.99(1.06,8.43) | 0.04 | 0.80(0.30,2.11) | 0.64 | 0.96(0.22,4.23) | 0.96 | 0.66 |  |
| Stroke |  |  |  |  |  |  |  |  | 0.51 |
| No | ref | 0.86(0.71,1.04) | 0.12 | 0.92(0.76,1.13) | 0.42 | 0.89(0.74,1.08) | 0.23 | 0.46 |  |
| Yes | ref | 0.58(0.20,1.70) | 0.31 | 0.43(0.13,1.43) | 0.16 | 0.32(0.07,1.40) | 0.13 | 0.14 |  |
| Coronary heart disease |  |  |  |  |  |  |  |  | 0.57 |
| No | ref | 0.85(0.71,1.03) | 0.10 | 0.90(0.75,1.09) | 0.28 | 0.87(0.72,1.05) | 0.15 | 0.33 |  |
| Yes | ref | 2.40(0.56,10.29) | 0.23 | 1.52(0.33, 6.88) | 0.58 | 0.97(0.24, 3.93) | 0.96 | 0.69 |  |
| Diabetes |  |  |  |  |  |  |  |  | 0.73 |
| No | ref | 0.87(0.70,1.08) | 0.19 | 0.92(0.73,1.15) | 0.46 | 0.86(0.70,1.06) | 0.16 | 0.29 |  |
| DM | ref | 1.03(0.57,1.85) | 0.92 | 0.95(0.52,1.73) | 0.86 | 1.15(0.64,2.08) | 0.63 | 0.63 |  |
| IFG | ref | 0.78(0.32,1.87) | 0.57 | 1.08(0.37,3.16) | 0.88 | 0.78(0.29,2.09) | 0.61 | 0.71 |  |
| IGT | ref | 0.58(0.13,2.51) | 0.45 | 0.54(0.15,1.94) | 0.33 | 1.27(0.34,4.72) | 0.71 | 0.49 |  |

The subgroup analyses were adjusted for all covariates except the stratification variable itself.
